# Experiences of connectivity and severance in the wake of a new motorway: Implications for health and well-being

**DOI:** 10.1016/j.socscimed.2017.11.049

**Published:** 2018-01

**Authors:** Amy Nimegeer, Hilary Thomson, Louise Foley, Shona Hilton, Fiona Crawford, David Ogilvie, M74 study team

**Affiliations:** a MRC/CSO Social and Public Health Sciences Unit, University of Glasgow, 200 Renfield Street, Glasgow, G2 3QB, United Kingdom; b Glasgow Centre for Population Health, Third Floor, Olympia Building, Bridgeton Cross, Glasgow, G40 2QH, United Kingdom; c MRC Epidemiology Unit & UKCRC Centre for Diet and Activity Research (CEDAR), School of Clinical Medicine, University of Cambridge, Box 285, Cambridge Biomedical Campus, Cambridge, CB2 0QQ, United Kingdom

**Keywords:** Natural experimental study, Road, Severance, Active travel, Transport, Qualitative, Motorway

## Abstract

The construction of new urban roads may cause severance, or the separation of residents from local amenities or social networks. Using qualitative data from a natural experimental study, we examined severance related to a new section of urban motorway constructed through largely deprived residential neighbourhoods in Glasgow, Scotland. Semi-structured and photo-elicitation interviews were used to better understand severance and connectivity related to the new motorway, and specifically implications for individual and community-level health and well-being through active travel and social connections.

Rather than a clear severance impact attributable to the motorway, a complex system of connection and severance was spoken about by participants, with the motorway being described by turns as a force for both connection and severance. We conclude that new transport infrastructure is complex, embedded, and plausibly causally related to connectedness and health. Our findings suggest the potential for a novel mechanism through which severance is enacted: the disruptive impacts that a new road may have on third places of social connection locally, even when it does not physically sever them. This supports social theories that urge a move away from conceptualising social connectedness in terms of the local neighbourhood only, towards an understanding of how we live and engage dynamically with services and people in a much wider geographical area, and may have implications for local active travel and health through changes in social connectedness.

## Highlights

• A complex system of connection and severance observed in relation to the motorway.
• New roads are complex, embedded, and plausibly causally related to health.
• Findings suggest potential for novel mechanism through which severance is enacted.
• Supports theories that conceptualise social connections beyond local neighbourhood.

## 1 Introduction

The neighbourhood built environment is an important influence on the health and wellbeing of residents. However, isolating the health impacts of specific changes in built environments (such as new transport infrastructure) is challenging as changes often occur within complex systems of associated micro- and macro-level changes. For example, building major new roads may promote health by improving access to employment, amenities and services, and contributing to economic development. However, new roads may also worsen the health and wellbeing of residents; for example by causing pollution, or impairing local connections (Boniface et al., 2015) and discouraging health-protective behaviours such as walking (Mindell and Karlsen, 2012). One such mechanism through which detrimental effects can occur is transport severance (Egan et al., 2003). Severance, a concept commonly associated with new transport infrastructure, is often defined as the creation of a physical and/or psychological barrier that divides people from local services or social connections within the community (Clark et al., 1991). As such, it has been shown to affect the health and wellbeing of residents through their use of local resources such as health services (Smith and Gurney, 1992; Mackett and Thoreau, 2015), their active travel (Smith and Gurney, 1992) and, although this has been less widely studied, influencing their social capital and feelings of community cohesion (Appleyard and Lintell, 1972, Hart and Parkhurst, 2011). These impacts have the potential to be particularly harmful in neighbourhoods with pre-existing low levels of resilience. Changes to transport infrastructure, however, do not take place in isolation, and it may be overly simplistic to view severance arising from such changes without reference to related micro- and macro-level factors and changes such as other infrastructure, economic climate, population change, and wider regeneration initiatives. In this paper, we examine the severance impacts of a new section of urban motorway constructed through largely deprived residential neighbourhoods in Glasgow, Scotland. We consider the effects of the motorway within a wider *system* of change as described by participants, and propose a novel mechanism through which severance is enacted in the form of the disruptive but non-bisecting impacts that new roads may have on third places of social connection within local areas.

## 2 Background

### 2.1 The relationship between new roads and severance

The creation of new roads may connect communities to each other, but can also form physical or psychological barriers, cutting one part of a community off from another. This ‘cutting off’ is also known as transport severance. Several definitions of severance are offered in transport and health literature and beyond (OECD (Organisation for Economic Co-operation and Development), 1973, Lee and Tagg, 1976, Clark et al., 1991, James et al., 2005, Anciaes et al., 2015, Anciaes et al., 2016). Severance has been classified as being either primary (restricting movement from one side of the infrastructure to the other) or secondary (indirectly or inconstantly contributing to severance effects); or as being physical in nature (preventing movement) or psychological (creating a perception of barriers) (Clark et al., 1991).

James et al. (2005) present a typology of the mechanisms through which severance is enacted by new roads. They suggest that this may occur through the creation of permanent physical barriers (physical infrastructure), temporary physical barriers (such as high traffic flows, which may be temporally patterned), omission barriers (lack of pedestrian infrastructure or crossing facilities), legal barriers (prohibitions against certain types of transport, e.g. no cycling on motorways), time barriers (weather-related, or fear of using some facilities at night), quality barriers (poor surfaces or lighting), attitudinal barriers (fear for personal safety leading to reluctance to travel), and information barriers (lack of knowledge about how to use or access facilities). For the purposes of this paper we will employ Clark et al. (1991) definition of severance, broadly encompassing any physical and/or psychological barrier that divides people from either local services or social connections within their communities, but will also make reference to James et al. (2005) *mechanisms* through which severance may be enacted.

### 2.2 The relationship between severance and health-related behaviours

Whether through physical or psychological means, transport-related severance may affect individual behaviour. If residents feel compelled to avoid a new road, this may lead to changes in their route or mode of travel (Smith and Gurney, 1992), their access to local health and other services (Smith and Gurney, 1992, Mackett and Thoreau, 2015), and their local social interactions (Appleyard and Lintell, 1972, Hart and Parkhurst, 2011). All three of these behaviours have been linked to health outcomes.

Although there is little evidence directly linking health outcomes with severance (Douglas et al., 2007) due to both a dearth of studies and the challenge of ascribing causality, there is evidence suggesting links in a plausible causal chain between severance and health-related behaviours (Mindell and Karlsen, 2012). For example, active travel is associated with positive health outcomes (Saunders et al., 2013) and therefore, transport infrastructure which curtails active travel through any of the mechanisms that James et al. (2005) describe may have negative health implications. Additionally, severance may affect residents’ access to local goods and services or the pleasantness of local streets or other sites as social spaces, and may therefore have an impact on social participation due to erosion of local connectedness (Appleyard and Lintell, 1972, Mindell and Karlsen, 2012) or may prevent residents from accessing amenities directly related to health, such as primary care or sports facilities. In short, if new infrastructure reconfigures social space in a way that severs social connections and networks, this is likely to have implications at both the individual (social support) and neighbourhood (social capital) level (Illich, 1974). Social capital itself has many definitions, but is often conceptualised as a benefit emergent from social connectedness at a neighbourhood/ecological level, and realised at both local and individual levels through social contacts, networks and participation (Coll-Planas et al., 2016). It can be enacted through bonding (cementing group cohesiveness) as well as bridging mechanisms (connecting those in disparate groups). Few studies have demonstrated strong evidence of a causal relationship between social capital and health (Coll-Planas et al., 2017, Durlauf, 2002), perhaps because of the complex nature of measuring or even defining such a multi-dimensional phenomenon (Rocco et al., 2014). However, there is evidence to suggest that social capital and its proxy social participation may be associated with outcomes such as decreased mortality (Kawachi et al., 1997), lowered blood pressure (Andersson, 1985) and better self-reported health via psychosocial mechanisms (Wilkinson, 1996).

### 2.3 Gaps in the literature

According to Anciaes et al. (2015), there is a lack of qualitative research exploring the possible severance effects of new transport infrastructure as experienced and perceived by local people. Such qualitative studies that do exist tend a) to come from diverse disciplines that may not overlap; and b) not to be published in academic journals, and to suffer from a lack of international dissemination. There are a few notable exceptions, including Hines' seminal 1994 study using interview and video data to examine the impact of traffic on pedestrian behaviour in Edinburgh, and Mindell et al.'s (2017) study of transport severance in England. A Department of Transport review (James et al., 2005) has suggested a need for more “*robust social research”* (p6) on the topic, and recent guidance from Mindell et al. (2017) suggests that qualitative evidence can and should be meaningfully integrated into mixed methods severance evaluations. In addition, previous studies have largely been focused on causally isolating the severance effects related to new transport infrastructure and have not investigated transport severance as existing within a complex system of physical and social connecting forces. This study endeavours to address these gaps in social research.

### 2.4 Background to the study

In 2011, a five-mile (8 km) extension to the M74 motorway was opened in Glasgow. Proponents of the road advocated that the motorway would enhance the wellbeing of local residents, at least partially through increased connectivity, whereas opponents claimed it would be detrimental, causing severance. There was considerable local opposition to the proposed motorway. This research forms the qualitative part of a mixed-method longitudinal natural experimental study of the health impacts of the motorway extension on local residents, with a particular focus on wellbeing, active travel and road traffic accidents. The overall study is described in detail elsewhere (Ogilvie et al., 2017).

**Fig. 1:**
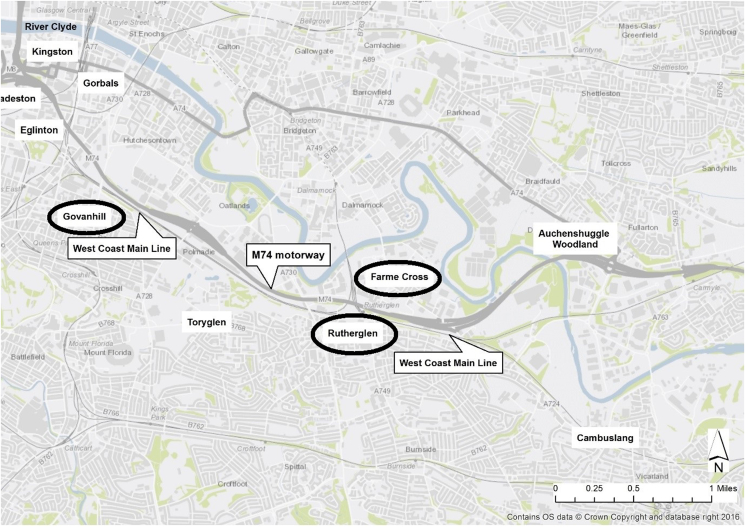
Map of Study Area with qualitative case-study communities circled.

The qualitative data collection reported in this paper was carried out with local residents (to capture micro-level change) and key informants (meso and macro-level change) in areas abutting the motorway extension. The analytical aims differed between the interviews with key informants and those with residents. The aim of the key informant interviews was to gain an overview of key environmental changes related to the new motorway. With resident interviews, the aim was to understand how participants perceived, experienced and used their neighbourhood, whether these had changed, and what role (if any) the new motorway had played in these changes. In this paper, we will present the combined findings from both resident and key informant interviews. For the resident interviews, we sampled participants from two neighbourhoods, Govanhill and Rutherglen (including Farme Cross) (Fig. 1). By viewing these two neighbourhoods as qualitative case study areas, we were able to observe differences in severance and connectivity at both neighbourhood and individual level, and consider the ways in which active travel and social cohesion related to health may have been affected by the motorway extension.

## 3 Methods

### 3.1 Research context

Glasgow is the fourth largest city in the United Kingdom (UK) with around 500,000 inhabitants in the core Glasgow City Council area. It is a city characterised by extremes of affluence and deprivation and has the lowest life expectancy in the UK (Hanlon et al., 2006). The M74 extension corridor runs through a combination of industrial and residential land in Glasgow's south side. Close to the western part of the extension lies Govanhill, a community characterised by economic deprivation, historic heavy industry, and successive waves of migration. Close to the eastern part of the extension lies Rutherglen, a historic royal burgh, with more mixed levels of housing than Govanhill but containing some of the most deprived datazones in Scotland according to the Scottish Index of Multiple Deprivation (Scottish Government, 2012). Rutherglen has also historically been a seat of heavy industry.

Along the extension corridor, the motorway mostly follows an existing railway line. Both the railway line and motorway extension run close to housing, in some areas within 50 m. Both case study areas had high levels of local traffic prior to motorway construction, and in both communities the majority of participants live on the same side of the motorway as some, but not all, of their local amenities. However, the physical character of the extension differs between the two areas. In Govanhill, residents experience three different segments of the new road infrastructure: a section of motorway running underneath existing roads, an elevated section, and a major motorway junction. In Rutherglen/Farme Cross, the motorway remains raised above its major connecting arteries. A motorway junction lies to the east of Rutherglen, removed from the main residential area.

### 3.2 Recruitment and sampling

#### 3.2.1 Resident interviews

A sample of 30 residents was purposively recruited from a larger survey sample of 1343 (described in more detail in Ogilvie et al., 2017). Recruitment letters were mailed out to 92 participants and followed up by phone call or email (depending on stated preference). Of these, 30 participants consented to an initial semi-structured interview (Table 1), with 12 opting for a follow-on photo-elicitation interview (Nykiforuk et al., 2011). Participants were evenly split between the neighbourhoods of Rutherglen and Govanhill, and all resided within 400 m of the motorway extension.

**Table 1:** Characteristics of resident participants.

Variable	Qualitative sample (n = 30)
	mean (SD)/%
Age (years)	52 (15)
% male	36
% home ownership	60
% car ownership	53
% working^a^	66
% with chronic condition	36
Perceived financial strain	
Quite comfortably off	13.3
Can manage without difficulty	13.3
Have to be careful with money	63.3
Find it a strain to get by	10.0
Years lived in local area	19.4 (18.3)

n – number; T – time point; SD – standard deviation.

a In paid employment (full or part-time), full-time student, or undertaking voluntary work.

#### 3.2.2 Key informant interviews

Key informants were recruited purposively based on their involvement with local groups. The groups were themselves identified by initial community engagement work undertaken by the Glasgow Centre for Population Health and the Scottish Community Development Centre. Additional key informants were identified by snowball sampling. Key informant participants represented a range of organisations including local community councils, local development groups, local charities, local housing associations, organisations involved in the planning and development of the motorway, and an anti-M74 protest group. Invitations and information sheets were emailed or mailed to 25 such organisations and followed up by either email or phone call. In cases where an individual key informant had already been identified by initial engagement, that key informant remained the point of contact for their group. Twelve participants consented to take part.

### 3.3 Data collection

Data were collected between May 2014 and April 2015 by AN. Resident and key informant interviews took place concurrently within the same time period.

#### 3.3.1 Resident semi-structured interviews

All resident participants undertook an initial semi-structured interview which was used to examine their general perceptions, experiences and uses of their local neighbourhood. Towards the end of the interview, if the motorway extension had not been mentioned it was raised by the researcher. The purpose of this approach was to understand which other factors were influencing the participants’ feelings of connectedness and their use and perceptions of the neighbourhood, and where the motorway extension might fit within a holistic view of neighbourhood change. The overall study was described as research examining traffic and health in Glasgow, and the motorway was not explicitly mentioned in any of the study documentation.

#### 3.3.2 Resident photo-elicitation interviews

Participants were given the choice of using their own digital camera/smartphone or a disposable camera. The subject of the photographs was negotiated between participant and researcher at the end of their first interview, but most participants chose to photograph a typical journey or to further illustrate points made during their interview. Participants returned their cameras or emailed their digital images to the researcher when they had finished, and negatives were developed if necessary to provide a set of images from each participant which formed the basis of discussion for their second interview. Participants were initially asked to sort the photographs in a way that was meaningful to them (e.g. by the order of a journey, themes, etc.) and then talk through them, explaining each photograph's significance. This was followed by a discussion of any photographs *not* taken, to better understand their experience of the process and how they chose to ‘frame’ the neighbourhood.

#### 3.3.3 Key informant interviews

Semi-structured key informant interviews were conducted in a variety of locations within Glasgow. These interviews were intended to better understand changes to the local environment related to the motorway extension, and were therefore much more specifically focused on the M74 than the resident interviews. The interviews for key informants focused on a number of a priori themes, including the impacts of the physical structure of the motorway itself and its associated engineering features, as well as its wider economic impacts. At the request of two key informants, the interview took place while walking through the space being discussed, rather than in a fixed location.

### 3.4 Ethics and informed consent

All participants were provided with an information sheet (plain language summary) and a verbal explanation of the study, and gave written informed consent. Ethical approval was obtained from the University of Glasgow's College of Social Sciences Ethics Committee (ref400130156).

### 3.5 Analytic approach

All interviews were transcribed verbatim by a transcription service and analysed thematically by AN. NVivo10 software was used to organise the data. Four out of nine transcripts from the pilot phase of the resident interviews, and four out of 12 key informant interviews were double coded by two additional researchers (HT and GF) to validate AN's emerging coding framework. Analysis of these datasets was carried out separately to begin with, and integrated later using a data matrix in pursuit of a more holistic understanding of neighbourhood change associated with the M74 extension.

To some extent, data collection and analysis were concurrent (in the form of reflective field notes), but further thematic analysis followed a five-step process: immersion in the data (rereading the transcripts whilst listening to the recordings), annotating transcripts according to key emerging topics/ideas, initial coding based on a priori codes from the topic guide and emergent codes, and amalgamating codes into themes (which also allowed us to identify which changes residents prioritised, and what they viewed as key drivers of change). To examine severance across the two datasets, the data were initially combined using the aforementioned matrix and approached using current definitions of severance as a physical or psychological barrier effect. More detail on the process of data analysis can be found in a supplementary file.

## 4 Findings

A complex system of connection and severance was described by participants, with the new motorway being described by turns as a force for both connection and, in some cases, severance. In this section, we will examine physical and psychological severance and connection as these were related to the motorway and other local factors, focusing in particular on their impacts on health-related behaviours such as active travel and on social connectedness.

### 4.1 Active travel

Findings suggested that a number of factors influenced the active travel of residents, some of which resonate with current understandings of severance, although many were not related to the motorway. We describe these impacts here briefly, as they are dealt with in more detail elsewhere (Ogilvie et al., 2017).

#### 4.1.1 Active travel after the motorway: impacts on walking and cycling

**Fig. 2:**
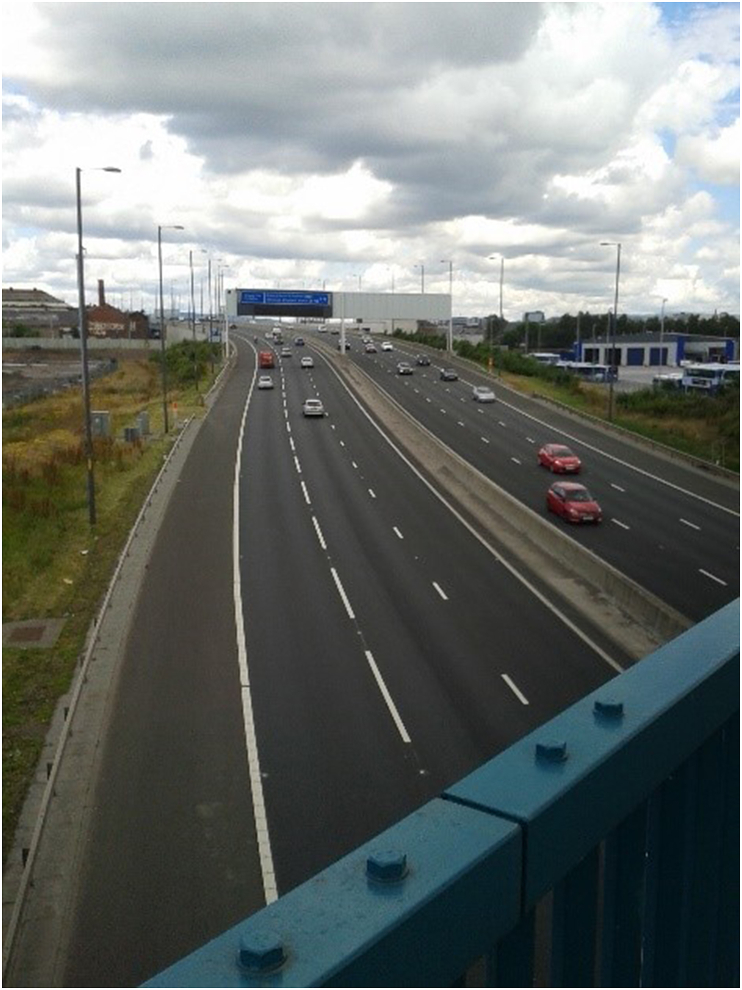
**M74 extension passing beneath an existing street in Govanhill,** Photograph ^©^ study participant and reproduced with permission.

**Fig. 3:**
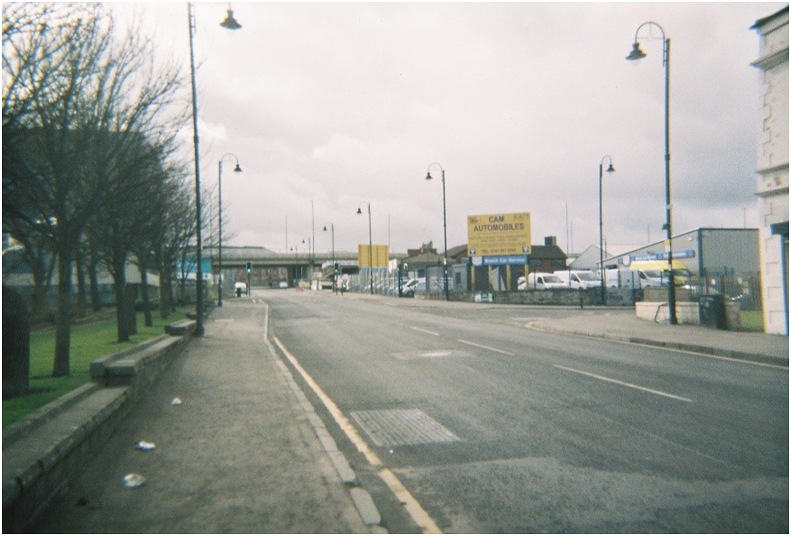
**M74 extension in the distance, passing over the road between Rutherglen and Farme Cross,** Photograph ^©^ study participant and reproduced with permission.

The motorway was associated by some participants with experiential changes in their active travel due to either the physical structure of the road or its impacts on local traffic. These changes ranged from positive to negative, depending largely on whether local traffic was perceived to have increased or decreased or whether the new infrastructure constituted a new barrier or overcame an existing one. In respect of active travel participants tended not to describe straightforward physical severance, i.e. changing their travel patterns to avoid a barrier presented by the motorway itself. However, some did describe changes to the experience or character of their active travel. Changes in noise, pollution, safety, and general changes to the aesthetic experience of walking were raised by a few residents both in positive and negative terms. Within the photo elicitation interviews, active travel routes pictured and described by residents included a mixture of routes that did and did not cross the motorway. Active travel journeys crossing over (Govanhill, Fig. 2) or under (Farme Cross/Rutherglen, Fig. 3) the motorway included journeys to access grocery stores, to commute to work, and to visit friends (although most journeys described were utilitarian in nature). The majority of these journeys were described as unchanged or positive, with the exception of two photo elicitation participants who described their journey as less pleasant due to noise or fumes:

Participant: It just makes it a little bit more noisier and, obviously, pollution from the petrol fumes … well, that's changed it.”

Male, 51–65, Rutherglen

Some changes to pedestrian infrastructure associated with the motorway were described as alleviating rather than causing severance. For example, a new footbridge over the motorway was considered by several residents to be an improvement over existing infrastructure that crossed the railway, creating a safer, more open, well-lit place to cross - in effect, mitigating an existing line of severance along the railway line:

I think they've actually improved the kinda bridge over … it's better now, than what it was. It used to be a kinda wee enclosed space like.

Male, 51–65, Govanhill

The new footbridge was cited by one participant as motivating their partner to walk more often when travelling between Govanhill and a nearby neighbourhood due to the improved personal safety associated with the new crossing point. This echoes findings from Sahlqvist et al. (2015) to the effect that pedestrian infrastructure that bridges existing severance features can act as “living landmarks” to encourage active travel. Additionally, in areas where traffic on surface roads had decreased, this was perceived as creating a more pleasant environment with the potential to increase local connectivity:

I think the motorway certainly would've changed the character [of the local area] because we don't get as much through traffic now … and it's much easier tae get out and about and crossing roads and things like that.

Male, 51–65, Rutherglen

Although overall numbers of cyclists were low amongst residents, changes to associated cycling infrastructure were not viewed as positively, especially around the motorway junction in Govanhill. This area was perceived by some as having become more hazardous due to increased traffic volume and changed configurations of cycle lanes, although participants did not report altering their cycling practices because of this:

where there used to be a bike lane there isn't one now. So yeah, that's changed and that's definitely more dangerous ‘cos the density of traffic has increased.

Key informant, local greenspace organisation

And in Rutherglen, one cyclist described changes to cycle routes near the M74 as inconsistent:

You're on the road, you're off the road, you're on the road you're off the road, so … it's just kinda badly thought.

Female, 36–50, Rutherglen

#### 4.1.2 Active travel beyond the motorway

Both physical and psychological severance effects on active travel were described by participants in relation to many factors other than the motorway, including busy or poorly designed local roads and poorly designed or maintained existing infrastructure, such as cycle lanes that were not clearly marked and a dimly lit narrow pedestrian tunnel under a main road. New, less effective street lighting was also described by one resident as creating a recurring temporary barrier to active travel, as were cars parked in bus or cycle lanes. On the whole, active travel was much more likely to be described as influenced by social factors than by those related to the motorway or traffic in general. Fear of crime was most frequently cited as a key factor in causing participants to either alter routes or avoid active travel entirely. This created a distinct line of severance through the community in Govanhill as residents actively avoided one particular road and encouraged family to do likewise.

Fear of crime also affected residents' perception of pedestrian infrastructure associated with the motorway. Rather than creating a barrier of omission (James et al., 2005), the perception of some residents (n = 4) was that the new pedestrian walkway in Govanhill helped to deter fear of crime through its well-lit paths and open structure. In other words, the ‘mitigation features’ associated with road building, such as pedestrian overpasses and tunnels mentioned, were considered to be successful or unsuccessful predominantly based on the degree to which they enhanced or detracted from a personal feeling of safety from crime. In this way, the new infrastructure helped to overcome existing severance barriers for some participants during active travel.

#### 4.1.3 Public transportation

Public transportation was important for the study respondents, and in general, a majority of residents in both areas considered their local neighbourhood to be well connected by railway and bus services. However, recent amendments to the schedules and locations of bus stops had caused difficulties for some people with reduced mobility:

there used to be a bus stop in the middle and they seem to have taken it away at this side walking down towards [neighbouring road]. There is one in the middle at the opposite side but it is really quite a long walk. There's no bus stop. I don't mind walking but it's for likes o’ the elderly, you know.

Woman aged 51–65 living in Rutherglen

they moved bus stops and it … [u]sed to stop outside the bookies but now they've taken it up to the cash and carry so it's quite a wee walk back for me and it's a bad pavement and a bad road and a big kerb, so that's awkward for me.

Woman aged 65 + living in Govanhill

In Govanhill, a small number of residents described changes as leading to more treacherous walks to the bus stop, and longer and more complex routes. In contrast, Rutherglen residents described the recent refurbishment of the train station as a positive development. Although residents did not associate these changes in public transport with the opening of the M74, for key informants the new motorway was seen as part of a broader programme investment whose causes and effects were difficult to disentangle.

### 4.2 Social interactions and community cohesion

#### 4.2.1 The motorway and social connection

Within the transport literature, there are two core dimensions of severance that relate to social networks and community connectivity: one is created by cutting residents off from local amenities such as shops and other services (limiting built capital), and the other by cutting people off from their local social contacts either physically or psychologically (social capital). With respect to the motorway and people's social networks, again, a mixed picture of connectivity and severance emerged from the data. For car users with dispersed social networks, the motorway was described as a way to connect with friends and family in other places:

I love it ‘cause I go down to the … Borders so it means I get on the motorway in five minutes where I used to have to drive up through Rutherglen, and take twenty minutes to get on the motorway. So I like it.

Female, 36–50, Govanhill

For non-car or non-motorway users, this benefit was not described except in cases where residents had relatives with cars who used the motorway to visit and connect with them. However, this was often discussed in terms of making the connection more convenient, rather than in facilitating more journeys (and therefore more incidences of connection).

#### 4.2.2 The motorway and connections to and through amenities

Participants also described the motorway as having both a connecting and severing effect with respect to amenities. For example, many residents with cars described the motorway as connecting them with work and leisure facilities:

I use it [the new motorway], I think, pretty much every day to go to my work in Queenslie, to go and visit family in Hamilton, to go to M&Ds [theme park], to go to Braehead or Silverburn [shopping centres]. So it's perfect for me, really.

Female, 20–35, Rutherglen

For those who did not own a car, had relatives that used the motorway, or who did drive but preferred not to use the motorway, the motorway was described as having either a neutral (“*it makes no difference to me at all”:* female 36–50, Rutherglen) or a negative impact:

Don't go out early in the morning at the rush hour because [motorway-adjacent road] is just completely jam packed … You used to just drive up tae the Asda [supermarket] in ten minutes. But since [the motorway opened], no. Even the light sequence is wrong, so it is. So, no it's completely, for people who don't use the motorway it is, it's a bit o’ a nuisance.

Female, 36–50, Govanhill

This echoes the findings of Rajé (2004), that motorways can cause severance for local drivers due to congestion at junctions and changes to local surface traffic. Residents often described their use of amenities as being embedded into wider journey patterns, for example picking up groceries on the way home from visiting family or work; in other words, their networks of connection to amenities and people extended beyond the local neighbourhood space.

Even so, several local amenities were identified as producing bonding (intra-group) or bridging (inter-group) social capitals. Local shops were considered by many to be important local spaces, both defining the local area for outsiders and providing places for social interactions. Several participants described them as important sites of casual local connection, with one resident describing them as the only place where he interacted regularly with anyone else from his local neighbourhood. Some residents did describe a declining use of local shops (particularly in Rutherglen), although this was ascribed not to the motorway but rather to a perception of declining quality of the shops; where there had been unique, small shops to entice local shoppers, now there was a preponderance of betting shops, money lenders, and takeaways. One resident expressed that pre-build, they had concerns that local shops would be affected by the motorway, which made them feel resistant. However, when negative impacts on shops failed to materialise, they felt acceptance towards the motorway's presence, even while acknowledging other potential drawbacks associated with it.

Neighbourhood places that facilitate such casual interactions are crucial to place-making and can do so deliberately or incidentally. Oldenburg (1989) and Putnam (1995) refer to such public spaces as ‘third places’ (first places being home and second places, work) in which people interact and which, among other things, are accessible, free to enter, and provide access to generally the same people on a regular basis. Local residents described local streets, social clubs, churches, shops, community hubs/social spaces created by local charities to create local links, and green spaces in this way.

Such interactions that occurred in these third places had implications for residents beyond the idea of ‘making friends’, because neighbourliness and being a ‘known face’ in the area were associated by some residents with greater feelings of local safety (producing both bonding and bridging social capital), and places where such contact occurs were therefore perceived as having some local importance. For example, for one resident, public parks were described as spaces where children (including those from different cultural backgrounds) could play together and form bonds:

So Govanhill Park … is regularly used … I think with the schools [close by]as well, that they are so multi-cultural, there's a lot of multi-cultural mixing in the park as well, so you'll often see not just the kids but the parents as well, so I think that's been great for the community that part.

Female aged 36–50, living in Govanhill

Green spaces in general were perceived by key informants as places where people from neighbouring local communities could come together to overcome social barriers, and adults could interact with other adults, animals (dogs) and children (what Blokland and Nast (2014) refer to as ‘contact assets’ in creating community familiarity). In one green space, this function was explicitly described as *“improv[ing] community cohesion”* (Key informant, local greenspace organisation). Residents described green spaces as particularly important within their community as ’healthy’ sites. Although most parks and greenspace sites in the study area were not affected by the new motorway, two greenspace sites directly abutting the motorway were described by key informants as being affected in terms of audible and visual disruption and polluting fumes:

It's had quite a big impact on the way, you know, the woodland is and the way people experience the woodland because it's no longer a place of tranquillity

Key informant, local greenspace organisation

This may be of especial concern because there is evidence to suggest that access to quality greenspace may play a role in mitigating health inequalities in deprived neighbourhoods (Mitchell and Popham, 2008). Some key informants described the disruptive effects of both the physical structure of the motorway and traffic noise on the experience of using these ‘restorative’ third places as particularly negative.

#### 4.2.3 Non-transport sources of severance and their impact on community coherence

Residents from both areas varied in their perceptions of their local community as being socially cohesive or not. While many positive descriptions of ‘neighbourliness’ were present in both communities, anti-social behaviours such as drinking and drug-taking were referenced in both areas as impacting the ‘welcomingness’ of local streets. In Govanhill, the most frequently cited cause of not wanting to engage in active travel was the perception of threat due to certain groups of people socialising on the streets outside their homes (while in and of itself not particularly transgressive, a practice that many residents described as intimidating – but that a handful of residents and key informants dismissed as an issue of perception).

Such factors as poor upkeep of streets, perception of ‘welcomingness’, and quality of shops appeared to be more significant factors in local peoples' willingness to walk locally than any changes to traffic or local infrastructure related to the motorway.

### 4.3 A note on community engagement with those living in extreme proximity

As previously mentioned, community engagement activities were undertaken for several purposes, one of which was to discuss emerging findings with local stakeholders including residents. Community engagement events also generated heterogeneous views with some people seeing the road as connecting and others as a severing force. It also emerged that residents living in extreme proximity to the motorway but who were not part of the original sampling frame for the study (due to their proximity to other roadways) experienced significant disruption in the form of noise, pollution (fumes and light), vibrations and impacts on their local travel. In one case, traffic exiting the motorway at speed on a local off-ramp caused local active travel to all but cease for their family. However, these accounts of extreme proximity produced an additional nuance related to severance; for those living very close, it was not just their experience of the community that was most affected, but their experience in their own home. By leaving their home and moving out into the community, the effects of the motorway lessened, so traditional severance definitions may not apply to them in the same way. In other words, for those in extreme proximity, the motorway was more impactful on their first space rather than any tertiary community gathering spaces. The community engagement was the only part of the study where these first-hand narratives of extreme proximity arose, adding to and complementing the qualitative and quantitative data collection.

Although these cases were not typical of the general experience of local community members in the wider motorway adjacent areas, the experience of those in extreme proximity must be acknowledged.

## 5 Discussion

In this study, we have examined severance as it relates not only to a new motorway, but also to the wider neighbourhood context of physical and social factors. Rather than clear impacts of severance and connectivity related to the new motorway, this study revealed a series of sometimes conflicting experiences by residents who were ostensibly exposed to the same ‘intervention’. This has been observed before in the case of new transport infrastructure (Kesten et al., 2015). The ways in which severance may be related to health and wellbeing have been comparatively little explored in transport research, and considering the ways in which changes to transport infrastructure can affect individual active travel and social interaction lies at the heart of furthering this understanding. Rather than experiencing changes to their routes of active travel as a result of the motorway, residents were more likely to describe changes in their *experience* of travelling actively. This echoes but also partially explains findings from the wider study that active travel did not change post-motorway in residents living near to the new motorway (Ogilvie et al., 2017). It should be acknowledged that such experiential changes to active travel have been found to have the cumulative potential to encourage or discourage active modes of travel in the future (Jacobsen et al., 2009), and this could translate to impacts on health in terms of levels of physical activity and opportunity for local social connection. This research has also shown that high quality mitigation features have the potential to overcome existing severance and, in so doing, may encourage active travel. It is clear that both severing and connecting factors observed here were influenced by spatial factors (depending on neighbourhood of residence and proximity to motorway), temporal factors (times of day when traffic is heavier), and individual factors (related to health, mobility, gender, length of residence, and perception of place).

This paper offers two important contributions to the literature around new transport infrastructure and severance. Firstly, it highlights the complex effects of new roads as facilitating both connection and severance in local communities, within a wider system of other, often unacknowledged, sources of connection and severance. Secondly, we suggest that the indirect impacts of new roads on the quality of gathering places (places central to the creation of bridging social capital), even when a direct barrier effect is not present, may be one mechanism through which local severance is enacted. More research is needed in this area.

The complex effect observed, in which the motorway acted as both connecting and severing force in the local community, supports social theories that urge a move away from conceptualising social connectedness in terms of only our local neighbourhood, towards an understanding of how we live and engage dynamically with services and people in a much wider geographical arena (Gustafson, 2001, Viry, 2012, Anderson, 2006).

**Fig. 4:**
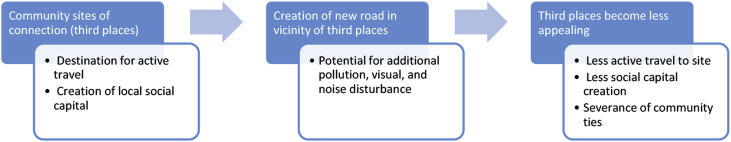
Creation of Severance through impact on third places.

Residents and key informants emphasised the importance of local physical spaces and these were often ones in which social interaction occurred either deliberately or serendipitously. Such spaces that emerged as important to participants in this study include shops, cafes, pubs, sports clubs, school gates, playing fields and green spaces. Walton (2014) terms such spaces vital places; locations that are both “important to and frequently-used by residents, and that are theoretically related to health through behavioural and/or social mechanisms” (p1). Traditional conceptualisations of severance focus on physical or psychological barriers created by traffic or road structures. We postulate that severance effects may be enacted by new roads through the degradation of third places, even if those places are not bisected by the road, nor do they or traffic associated with them create any form of barrier between residents and the third place. Emerging data about the sensory impact of the new road on the quality of some of adjacent greenspaces, *even when the motorway did not physically bisect these spaces*, may suggest a newly identified mechanism through which transport severance may potentially be enacted. When the quality of these spaces is compromised by new transport infrastructure, this may be sufficient to make such spaces lose the unique qualities (such as calm, quiet and ‘naturalness’ in the case of local green space) that make them attractive as local gathering spaces, thus indirectly causing a social severance effect (Fig. 4).

The extent to which these spaces are or are not affected by the new road may be significant in terms of local experiences of a severance effect, and this may affect ‘acceptance’ of the new infrastructure; however further research is needed to investigate this. Future impacts on these spaces may have implications for health, as our participants described them variously as contributing to individual wellbeing as well as the creation of a sense of community; as bridging gaps between physically and psychologically separate areas; and as having the potential to break down perceived divisions based on ethnic and cultural differences. The ways in which the motorway impacts on links with amenities or social networks, including through the potential impact on third places in which connections are built, has implications for both bonding and bridging forms of social capital, both of which have been linked to positive health outcomes (Coll-Planas et al., 2017, Rocco et al., 2014). There is also evidence, albeit limited, that social capital and connectivity can provide a ‘buffer effect’ on health inequalities (Uphoff et al., 2013), which suggests that interventions with potential impacts on connectivity in already disadvantaged neighbourhoods merit greater evaluative attention in this respect, especially since the wider study found that those living in close proximity to the new motorway experienced a slight reduction in overall wellbeing (Ogilvie et al., 2017).

Disentangling the severance and connectivity impacts of new transport infrastructure can be a complex undertaking, as the impacts are embedded within a wider system of local connection and severance. This study has also shown that a number of local factors, unrelated to the motorway, influenced how people interpreted and used the local community; and that these factors, including fear of crime, were seen to cause severance-like impacts and changes to local active travel over a similar timescale to the construction of the motorway. Considering other causes of severance within the neighbourhood allowed us to develop a clearer sense of plausible causal associations related to changes in the built environment.

There are limitations to this study. Firstly, the qualitative sample described here does not contain residents residing in ‘extreme proximity’ to the motorway, who we know from community engagement work have experienced more severe impacts than those of the wider population. Additionally, in terms of transferability (Lincoln and Guba, 1985), it is worth reiterating that this study took place in an area with pre-existing severance from a railway line, high levels of pre-existing traffic, an industrial past, and socioeconomic deprivation, which may well have influenced local perceptions about the acceptability of the new road. The topic of generalisable causal inference is a key issue in the field of environmental interventions, and is explored in the report on the wider mixed-method natural experimental study, of which the current analysis comprises one part (Ogilvie et al., 2017).

## 6 Conclusions

The building of a new motorway was observed to act as both a connecting and severing force on the local community. Perceptions of the motorway were variable from the neighbourhood level down to the individual level. For some people, the new road was perceived as facilitating active travel and social connectedness due to the provision of well-designed pedestrian infrastructure, lessening local traffic, and improved road connections for those with dispersed social networks and access to cars. For others, the new road was perceived as degrading the local experience of active travel and negatively affecting local spaces of connection. By viewing motorway-related severance and connectivity as part of a wider system, social changes such as demographic changes, and changes to individual and collective perceptions of personal safety were seen to act as sources of severance and made up part of the complex contextual mix that impacted how residents received the motorway. Background factors (including pre-existing severance from an existing railway line and high levels of pre-existing traffic) also highlight the importance of local context in the experience of severance.

To summarise, new transport infrastructure is complex, embedded, and plausibly causally related to connectedness and health. Social connectivity and active travel both have plausible links with health outcomes. In this paper, outcomes related to social connectivity and active travel have been attributed to the new road, but in more complex ways than previously described. This study has shown a mixture of impacts brought about by transport infrastructure but further study is recommended to explore the concept of severance via the impact of transport infrastructure on third places that facilitate local connection. It has also provided evidence for policymakers and planners that investing in high quality pedestrian infrastructure to overcome severance is worthwhile for residents.

Accordingly, our data suggest but do not confirm a novel theoretical mechanism through which severance is enacted that requires further exploration: the disruptive but non-bisecting impacts that new roads may have on adjacent third places of social connection within local areas, such as greenspaces. We also suggest that the complex interplay between social factors and perceptions of the new road found in our data, may indicate that building large motorways through areas of relative deprivation requires careful thought as to the social justice implications of such built environment change.
