# Supplementary material for: Experiences of connectivity and severance in the wake of a new motorway: Implications for health and well-being
Source: Soc Sci Med. 2018 Jan;197:78–86. doi: 10.1016/j.socscimed.2017.11.049 (PMC5777829; doi:10.1016/j.socscimed.2017.11.049)
Supplement: Online data [file mmc1.docx]

Appendix A: Data Analysis

**Thematic Analysis Process**

Resident Interviews Key Informant Interviews

1. Immersion in the data

2. Annotation of transcripts with key emergent topics/ideas

3. Initial coding of a priori themes (from topic guide) and emergent ideas

4. Double coding and agreement of key codes followed by recoding of entire dataset

5. Amalgamation into themes


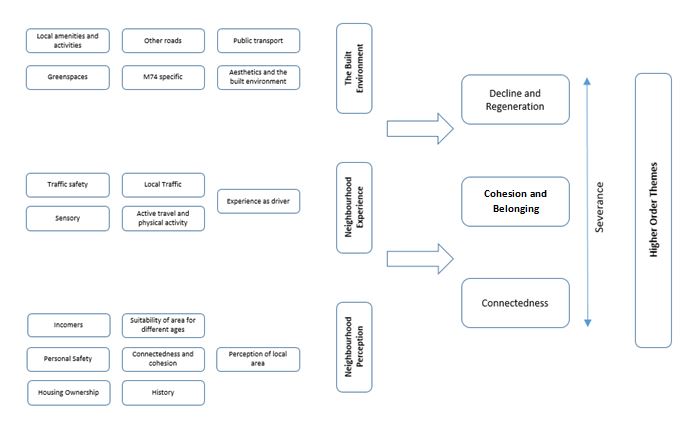


Code to theory data model

Resident Interviews


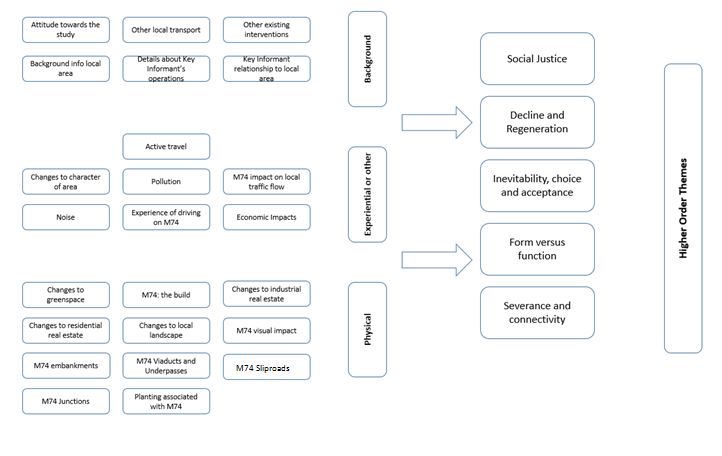


Code to theory data model

Key Informant Interviews

**Bringing the datasets together**

In order to bring together the two datasets, data from both Key Informant and Resident interviews related to key research questions (including severance) were gathered in data matrix. To analyse data with relevance to community severance we interrogated the new combined dataset using current definitions of physical and psychological severance and data related to:

- Perception of local area
- Physical or psychological barriers
- Local and non-local travel and active travel
- Local and non-local social relationships
- Use of local spaces and amenities
- Perceptions of connectedness with neighbouring communities

First looking for changes in the above related to the motorway, then changes in the above related to other sources.
